# Metacognitive Prediction of Creative Performance in Divergent Thinking

**DOI:** 10.1002/pchj.70126

**Published:** 2026-10-26

**Authors:** Tie Sun, Xingxu Xie, Yabing Sun, Jiayue Lin, Yuxuan Yang, Jing Luo

**Affiliations:** ^1^ Joint Education Institute of Zhejiang Normal University and University of Kansas, Zhejiang Normal University Jinhua China; ^2^ School of Psychology, Capital Normal University Beijing China

**Keywords:** creative metacognition, creativity assessment, divergent thinking, metacognitive prediction, semantic retrieval

## Abstract

Understanding how individuals monitor and predict their own creative performance is essential for advancing theories of creative metacognition. Despite extensive research on metacognitive predictions in memory and problem solving, little is known about whether people can accurately anticipate their performance in divergent thinking tasks. The present study examined prospective metacognitive prediction accuracy in the Alternative Uses Task (AUT), focusing on fluency, flexibility, and originality. Thirty‐two participants first ranked 10 everyday objects according to how well they believed they would perform on each AUT item and subsequently completed a verbal AUT for the same objects. Creative performance was independently scored by trained raters, and prediction accuracy was quantified using Gamma correlations between predicted and actual performance rankings. Results showed above‐chance prediction accuracy for fluency and flexibility, but not for originality. These findings suggest that individuals can prospectively monitor aspects of divergent thinking supported by semantic accessibility and category‐based search, but may have difficulty anticipating originality, which may depend more strongly on remote association and representational transformation. The study contributes to research on creative metacognition by showing that metacognitive prediction accuracy in divergent thinking is dimension‐specific rather than uniformly accurate or inaccurate.

## 1 Introduction

Creative thinking plays a fundamental role in problem solving, innovation, learning, and adaptation across a wide range of real‐world contexts. Among different facets of creativity, divergent thinking (DT)—the ability to generate multiple, varied, and original ideas—has long been regarded as an important indicator of creative potential (Guilford 1967). The Alternative Uses Task (AUT), in which individuals generate unconventional yet appropriate uses for common objects, is one of the most widely used tasks for assessing DT. Although extensive research has examined the cognitive processes underlying DT performance, less is known about how individuals monitor and predict their own creative performance before engaging in such tasks.

This issue is closely related to the broader construct of creative metacognition or meta‐creativity. In the creativity literature, creative metacognition has been conceptualized as more than a simple extension of general metacognition to creative tasks. Kaufman and Beghetto (2013) proposed that creative metacognition involves the integration of creative self‐knowledge and contextual knowledge, which allows individuals to judge when, where, and how creativity should be expressed. More recently, Lebuda and Benedek (2023) proposed a systematic framework of creative metacognition that distinguishes among metacognitive knowledge, metacognitive monitoring, and metacognitive control in creative cognition. According to this view, creative metacognition includes not only the assessment of one's creative performance but also the regulation of creative strategies, effort allocation, and idea evaluation. In the present study, however, we focus on one specific component of creative metacognition: prospective metacognitive prediction accuracy, namely, the extent to which individuals can predict how well they will perform on different DT items before actually completing the task. This operational definition allows us to examine creative metacognition at the level of task‐specific prospective monitoring, without assuming that the present findings generalize to all aspects of meta‐creativity.

Metacognitive prediction has been widely studied in memory and problem‐solving contexts. Research on judgments of learning (JOLs), feeling‐of‐knowing (FOK), and other prospective monitoring processes has shown that individuals often rely on heuristic cues, such as familiarity, processing fluency, and subjective accessibility, to estimate future performance (Koriat 1997; Nelson 1984; Nelson et al. 1984). These cue‐utilization mechanisms can support accurate predictions when the cues are diagnostic of later performance. For example, in memory tasks, subjective accessibility is often informative because items that feel easier to process are more likely to be remembered or recognized later (Koriat 1997). In contrast, prediction accuracy is typically lower in insight problem solving, where solutions often emerge discontinuously through representational change, constraint relaxation, or restructuring (Knoblich et al. 1999; Metcalfe 1986; Weisberg 1995). Because these processes are difficult to access before solution discovery, individuals often show poor correspondence between their predictions and actual success in insight‐like creative problem solving (Metcalfe 1986; Storm and Hickman 2015).

DT occupies a theoretically important intermediate position between these two types of tasks. On the one hand, DT resembles memory tasks because idea generation relies heavily on semantic memory retrieval. In the AUT, individuals often begin by retrieving common and accessible uses before moving toward less typical associations, a pattern consistent with spreading activation models of semantic memory and associative theories of creativity (Collins and Loftus 1975; Mednick 1962). Empirical studies of AUT performance also suggest that early idea generation is strongly shaped by semantic retrieval and search processes (Beaty and Silvia 2012; Gilhooly et al. 2007; Smith et al. 2013). These retrieval‐based processes may provide internal cues that support prediction. On the other hand, DT also shares features with creative problem solving and insight, especially when originality is required. Original ideas often depend on moving beyond dominant associations, recombining object features, or restructuring the representation of an object's function (Gilhooly et al. 2007; Knoblich et al. 1999). These processes may be less accessible to conscious monitoring before the task is performed. Therefore, DT may not be globally predictable or unpredictable; rather, prediction accuracy may differ across its component dimensions.

This distinction is important because DT is typically evaluated along multiple dimensions, including fluency, flexibility, and originality (Guilford 1967). Fluency refers to the number of ideas generated, and flexibility refers to the diversity of conceptual categories represented in those ideas. Both dimensions are closely linked to semantic accessibility, associative search, and category‐based retrieval (Beaty and Silvia 2012; Gilhooly et al. 2007; Stephens and Krebs 1986). When individuals view an AUT object, they may be able to sense whether many uses or many categories of uses are readily available. These subjective cues may support accurate prediction of fluency and flexibility. In contrast, originality refers to the novelty or uncommonness of generated ideas. Original responses are often produced later in the ideation sequence and may require remote association, conceptual recombination, or representational transformation (Beaty and Silvia 2012; Gilhooly et al. 2007; Mednick 1962). Because these processes are less directly cued by initial feelings of accessibility, individuals may have difficulty predicting their originality in advance.

Recent work on creative metacognition further supports the need to distinguish between general creative self‐assessment and task‐specific prediction accuracy. For example, Jiang et al. (2023) found that individuals with higher metacognitive ability showed better performance in divergent and convergent thinking tasks, suggesting a meaningful association between metacognition and creative cognition. However, such findings do not directly address whether individuals can predict, before performing a DT task, which specific items will lead to higher fluency, flexibility, or originality. Similarly, conceptual and empirical work on metacognitive feelings in the creative process suggests that subjective feelings may inform idea evaluation and selection, but these feelings may not always provide accurate cues for originality or idea quality (Puente‐Díaz et al. 2021; Puente‐Díaz 2023). Thus, the present study extends prior work by examining prospective, item‐level prediction accuracy across specific dimensions of DT.

The creative cliff illusion provides an additional reason to expect inaccurate prediction of originality. Lucas and Nordgren (2020) showed that people often believe their creativity will decline over the course of an ideation session, even though actual creativity often remains stable or increases. This illusion suggests that individuals may mistake decreasing fluency or increasing subjective difficulty for decreasing creativity. In the context of the AUT, such a bias may lead individuals to rely on the ease with which common ideas come to mind when predicting performance. This strategy may be useful for predicting fluency and flexibility, but it may be misleading for originality, which often emerges after the most accessible responses have been exhausted (Beaty and Silvia 2012; Lucas and Nordgren 2020). Therefore, the creative cliff illusion is directly relevant to the present study because it suggests that fluency‐based cues may systematically bias metacognitive predictions of originality.

Despite growing interest in creative metacognition, relatively little work has directly examined whether individuals can prospectively predict their performance in DT tasks. Existing research has more often focused on general creative self‐beliefs, post‐task self‐evaluations, metacognitive feelings, or broad metacognitive ability in relation to creative performance (Jiang et al. 2023; Kaufman and Beghetto 2013; Lebuda and Benedek 2023; Puente‐Díaz et al. 2021). These studies have advanced understanding of meta‐creativity, but they do not directly answer whether individuals can predict which AUT items will elicit better fluency, flexibility, or originality before generating responses. Accordingly, the present study addresses a more specific question: whether prospective metacognitive prediction accuracy differs across distinct dimensions of DT.

The present study examined this question using a within‐subject AUT design. Participants first viewed 10 everyday objects and ranked them according to how well they believed they would perform on each object. They then completed a verbal AUT for the same objects. Creative performance was scored independently in terms of overall creativity, fluency, flexibility, and originality, and prediction accuracy was quantified by the association between predicted rankings and actual performance rankings. Based on the theoretical distinction between accessibility‐based and transformation‐based processes, we proposed two hypotheses.

*Participants would show above‐chance prediction accuracy for fluency and flexibility, because these dimensions are supported by semantic accessibility and associative search.*

*Participants would not show reliable prediction accuracy for originality, because originality depends more strongly on remote association, representational transformation, and late‐emerging idea generation, which are less accessible to prospective monitoring.*

## 2 Method

### 2.1 Participants

Thirty‐four participants (10 males, 24 females; mean age = 22.50 years, SD = 1.58) participated in this experiment. Participants were undergraduate or graduate students recruited from Capital Normal University, and their academic backgrounds were recorded to describe the sample characteristics. None of the participants had prior experience with divergent‐thinking experiments. All participants were right‐handed, reported no history of neurological or psychiatric disorders, and had normal or corrected‐to‐normal vision. Two participants were excluded because they failed to complete the full ranking of all 10 AUT objects within the 30‐s prediction window. Specifically, their rankings were incomplete or contained repeated or missing ranks after the time limit, making it impossible to compute valid item‐level prediction accuracy. Thus, the final sample consisted of 32 participants. The study was approved by the Ethical Committee of the School of Psychology, Capital Normal University, and all participants provided written informed consent before the experiment.

### 2.2 Experimental Design

#### 2.2.1 Materials

In the Alternative Use Task (AUT), participants were presented with common objects (e.g., a brick) and instructed to generate as many unusual yet appropriate uses as possible within a specified time frame. Ten objects were selected for this experiment (*Scissors*, *Chopstick*, *Sheet*, *Ballpoint Pen*, *Vase*, *Watch*, *Backpack*, *Key*, *Newspaper*, and *Helmet*). The objects were selected to be common everyday items that were familiar to university students and capable of eliciting multiple possible uses. They were chosen to vary in typical function while avoiding highly specialized or obscure objects. However, they were not formally normed for familiarity, semantic association breadth, or visual complexity; this issue is acknowledged as a limitation.

#### 2.2.2 Procedure

This experiment was administered online via Tencent Meeting and PowerPoint. Participants completed the task individually and were instructed to stay in a relatively private and quiet environment, keep their microphone on throughout the task, and avoid using external aids such as notes, search engines, or communication tools. The experimenter interacted with each participant one‐to‐one throughout the entire procedure, monitored task engagement in real time, presented all task materials via PowerPoint, and recorded participants' verbal responses during the AUT stage. The verbal responses were subsequently transcribed by trained research assistants and checked against the original recordings to ensure transcription accuracy.

The experimental procedure is shown in Figure 1. The formal experiment consisted of two stages: the metacognitive prediction stage and the formal AUT stage. Before entering the formal experiment, participants were informed about the experimental procedure and the requirements of the AUT via PowerPoint. After participants fully understood the experimental procedure, the metacognitive prediction stage began. Participants saw the names of 10 everyday objects simultaneously and were asked to rank the objects according to their predicted creative performance, from 10 (highest expected performance) to 1 (lowest expected performance) within 30 s. They were explicitly instructed to make only a prospective prediction and not to generate actual uses during this stage.

**FIGURE 1:**
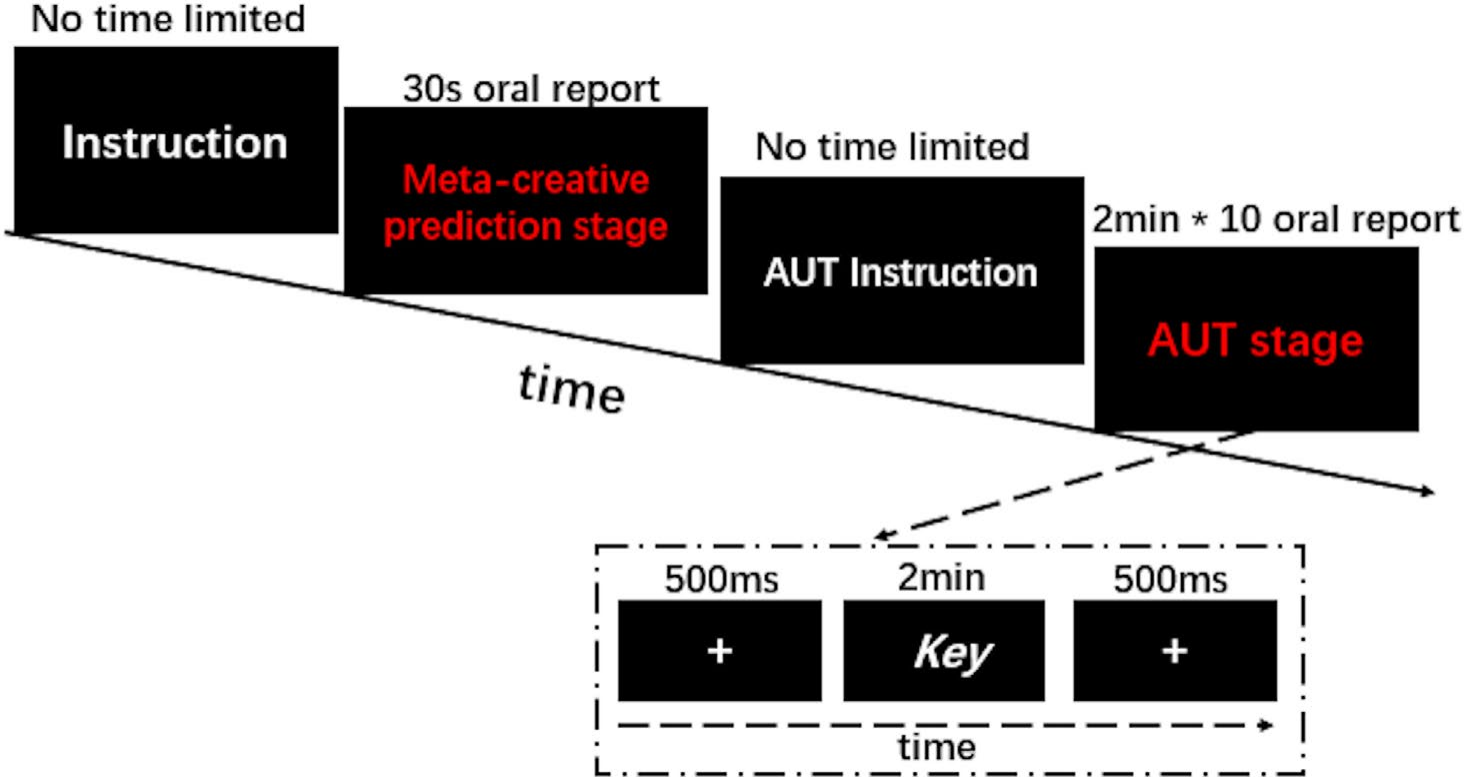
Experimental procedure and timeline. Participants first completed a 30‐s metacognitive prediction ranking for 10 AUT objects, followed by the formal AUT stage. In the AUT stage, each object was presented one at a time, and participants verbally generated unconventional yet appropriate uses within 2 min. A 500‐ms blank interval was inserted between consecutive objects.

The 30‐s prediction window was determined based on a small pilot check and post‐task interviews. The goal was to provide sufficient time for participants to rank the 10 objects while minimizing the opportunity to covertly generate AUT responses. In the formal study, participants were again reminded not to generate uses during the prediction stage. After the experiment, participants were asked whether they had attempted to generate uses during the prediction period.

After the prediction stage, the formal AUT stage began. The 10 object names were presented one by one in random order. When an object name appeared, participants verbally reported as many unconventional yet appropriate uses as they could within 2 min. A 500‐ms blank screen interval was inserted between consecutive objects.

#### 2.2.3 Data Analysis

Participants' creative performance was evaluated independently by three trained psychology students using the verified transcripts of verbal AUT responses. Before scoring, all responses were screened for validity. Appropriate responses were defined as uses that were physically possible or conceptually plausible for the target object. Unconventional responses were defined as uses that went beyond the object's typical or canonical function. Responses that were impossible, irrelevant to the object, repeated within the same object, or too ambiguous to interpret were excluded before scoring.

For each participant, the scoring process consisted of two steps. First, raters ranked the participant's AUT performance across the 10 objects according to overall creative level. Second, each valid response was scored according to standard AUT scoring procedures (Guilford 1967). Creative performance for each object was evaluated along three dimensions: fluency, flexibility, and originality. Fluency was calculated as the total number of valid responses generated for each object. Flexibility was calculated as the number of distinct conceptual categories represented by the responses. Originality was rated on a 5‐point scale based on the relative occurrence frequency of each response in the full response pool. Responses that appeared more frequently across participants were assigned lower originality scores, whereas rarer responses were assigned higher originality scores. Specifically, 1 indicated a highly frequent and less original response, whereas 5 indicated a rare and highly original response. Because the scoring procedure relied on relative frequency‐based judgment rather than fixed percentage thresholds, this issue is acknowledged as a limitation in the Section 4.

Goodman–Kruskal's Gamma correlations were computed between each participant's predicted ranking of the 10 AUT objects and the corresponding actual performance ranking for each creativity indicator. Gamma correlation was used because both predicted performance and actual performance were represented as ordinal rankings across the 10 AUT objects for each participant. Compared with Spearman's rank correlation, Gamma is particularly suitable for ordinal association when tied ranks may occur because it evaluates the balance of concordant and discordant pairs while excluding tied pairs. This feature was relevant to the present study because actual AUT performance scores, especially fluency, flexibility, and originality scores, could produce ties across objects. This approach is also consistent with prior metacognitive monitoring research using rank‐based judgments (Nelson et al. 1984). For each participant, Gamma coefficients were calculated separately for overall creativity, fluency, flexibility, and originality. The resulting Gamma coefficients were then compared with 0 using one‐sample *t*‐tests, where coefficients significantly greater than 0 indicated above‐chance prediction accuracy.

## 3 Results

Before the main analyses, we first checked the validity of the metacognitive prediction rankings. Because the prediction stage was implemented as a verbal ranking task rather than a computerized trial‐by‐trial response task, precise reaction times for each ranking decision were not recorded. However, all included participants completed a full and valid ranking of the 10 AUT objects within the 30‐s prediction window, with no missing or repeated ranks. Thus, the prediction rankings of the 32 included participants were considered valid for subsequent Gamma correlation analyses. To assess inter‐rater reliability, we computed intraclass correlation coefficients (ICCs) for the ratings across the 10 AUT objects and the three scoring dimensions. The ICCs for the 10 objects ranged from 0.757 to 0.936, indicating acceptable to excellent inter‐rater reliability. The ICCs for the three dimensions ranged from 0.555 to 1.00, indicating moderate to excellent reliability. Therefore, the three raters' scores were averaged for subsequent analyses.

The results of metacognitive prediction accuracy are shown in Table 1. For overall creativity, the mean Gamma correlation between participants' predicted rankings and actual performance rankings was significantly greater than zero, *M* = 0.127, SD = 0.281, *t*(31) = 2.555, *p* = 0.016, Cohen's *d* = 0.639. This result indicated that participants showed above‐chance prediction accuracy for their overall creative performance.

**TABLE 1:** Gamma correlation coefficients between metacognitive prediction rankings and actual performance rankings.

Meta‐creative prediction	Gamma *M*	SD	*t*	*p*	Cohen's *d*
Overall Creativity	0.127	0.281	2.555	0.016	0.639
Fluency	0.189	0.282	3.776	0.001	0.948
Flexibility	0.137	0.278	2.800	0.009	0.697
Originality	−0.122	0.390	−1.765	0.087	−0.442

*Note:* Gamma coefficients were compared with zero using one‐sample *t*‐tests. Positive Gamma values indicate above‐chance prediction accuracy; negative values indicate a tendency for predicted rankings to be inversely associated with actual performance rankings.

We then examined prediction accuracy separately for the three dimensions of DT. Prediction accuracy was significantly above chance for fluency, *M* = 0.189, SD = 0.282, *t*(31) = 3.776, *p* = 0.001, Cohen's *d* = 0.948, and flexibility, *M* = 0.137, SD = 0.278, *t*(31) = 2.800, *p* = 0.009, Cohen's *d* = 0.697. In contrast, prediction accuracy for originality was not significantly greater than chance and showed a negative trend, *M* = −0.122, SD = 0.390, *t*(31) = −1.765, *p* = 0.087, Cohen's *d* = −0.442.

This pattern suggests that the significant prediction accuracy for overall creativity should not be interpreted as evidence that participants could accurately predict all components of creative performance. Rather, the overall creativity effect appeared to be primarily consistent with above‐chance prediction of fluency and flexibility, whereas originality showed no reliable positive prediction accuracy. Thus, metacognitive prediction accuracy in the AUT was dimension‐specific: participants were able to predict performance dimensions associated with idea quantity and category diversity, but not originality.

## 4 Discussion

The present study examined whether individuals can prospectively predict their own DT performance in the AUT. Results showed above‐chance prediction accuracy for overall creativity, fluency, and flexibility, but not for originality. This pattern suggests that metacognitive prediction in DT is dimension‐specific. Individuals appeared to predict performance more accurately for dimensions closely related to idea quantity and category diversity, whereas prediction accuracy did not reliably extend to originality.

### 4.1 Dimension‐Specific Metacognitive Prediction in Divergent Thinking

The significant prediction accuracy for fluency and flexibility is consistent with cue‐utilization accounts of metacognitive monitoring. According to this perspective, individuals rely on internal cues such as familiarity, processing fluency, and subjective accessibility when estimating future performance (Koriat 1997; Nelson 1984). In the context of the AUT, fluency and flexibility are closely related to the accessibility and breadth of semantic associations. When individuals view a common object, they may be able to sense whether many possible uses or many conceptual categories are readily available. These subjective impressions may provide diagnostic cues for predicting the number and diversity of ideas that can later be generated.

This interpretation is also consistent with cognitive accounts of DT. Prior research suggests that early idea generation in the AUT is strongly shaped by semantic retrieval, associative search, and category‐based exploration (Beaty and Silvia 2012; Gilhooly et al. 2007; Smith et al. 2013). Fluency reflects the quantity of generated ideas, and flexibility reflects the diversity of conceptual categories. Both dimensions may therefore be supported by relatively accessible internal cues. Thus, the present findings suggest that individuals can prospectively monitor some aspects of DT when those aspects depend on processes that are relatively available to conscious awareness.

However, this conclusion should be interpreted carefully. The present study did not directly measure semantic accessibility, associative search strategies, or participants' subjective cues during prediction. Therefore, the accessibility‐based explanation remains theoretically plausible but indirect. Future research could include measures of perceived familiarity, subjective ease of idea generation, or self‐reported prediction cues to test whether these variables mediate prediction accuracy for fluency and flexibility.

### 4.2 Why Originality Was Not Reliably Predicted

In contrast to fluency and flexibility, originality was not predicted above chance. This finding is consistent with the view that originality depends on processes that are less accessible to prospective metacognitive monitoring. Original ideas often require individuals to move beyond dominant semantic associations, reconsider the typical function of an object, combine distant concepts, or restructure object representations (Gilhooly et al. 2007; Mednick 1962). These processes resemble, at least in part, the representational change and restructuring involved in insight problem solving, where prospective prediction accuracy is often poor (Metcalfe 1986; Storm and Hickman 2015). From this perspective, individuals may have difficulty predicting originality because the processes that produce original responses are not fully available before the task is performed.

Notably, prediction accuracy for originality showed a negative, though non‐significant, trend. This pattern suggests that participants may not merely have failed to predict originality accurately; they may have tended to rank some objects as promising when those objects later produced less original responses. One possible explanation is that participants relied on initial accessibility cues when making predictions. Objects that seemed easy to generate uses for may have been ranked highly, but such ease may have reflected the availability of common uses rather than the potential for rare or original responses. In other words, subjective fluency may have served as a misleading cue for originality.

This interpretation is consistent with the creative cliff illusion. Lucas and Nordgren (2020) showed that people often believe creativity declines as ideation continues, even though originality may remain stable or increase over time. The illusion suggests that people may confuse declining fluency or increasing effort with declining creativity. In the present study, participants may have relied on a similar fluency‐based heuristic when predicting their AUT performance. Such a heuristic could support accurate prediction of fluency and flexibility but become non‐diagnostic, or even misleading, for originality, which often emerges after common responses have been exhausted (Beaty and Silvia 2012; Lucas and Nordgren 2020). Thus, the negative trend for originality may reflect a systematic bias in which objects that feel easy at first are expected to produce creative performance, even if they mainly elicit conventional responses.

### 4.3 Overall Creativity and the Originality Discrepancy

The significant prediction accuracy for overall creativity also requires careful interpretation. Although overall creativity is often conceptually associated with originality, overall judgments in AUT scoring may also be influenced by fluency, flexibility, and other task‐performance cues. DT performance is commonly assessed through multiple indices, including fluency, flexibility, originality, and sometimes elaboration, and these indices do not necessarily capture the same cognitive processes (Guilford 1967; Reiter‐Palmon et al. 2019). Moreover, different scoring methods for the AUT can yield different patterns of association with cognitive, behavioral, and neural measures, suggesting that “overall creativity” should not be treated as equivalent to originality alone (Reiter‐Palmon et al. 2019; Vartanian et al. 2020). In the present study, prediction accuracy was above chance for fluency and flexibility but not for originality. This pattern suggests that the significant overall creativity effect should not be interpreted as evidence that participants could accurately predict all components of creative performance. Rather, the overall creativity effect may have reflected participants' sensitivity to more accessible performance cues, such as the expected number and diversity of ideas.

This interpretation may help explain the apparent discrepancy between significant prediction accuracy for overall creativity and non‐significant, negatively trending prediction accuracy for originality. Overall creativity ratings may capture a composite impression of performance, incorporating idea quantity, category diversity, and perceived novelty. This is consistent with the broader AUT assessment literature showing that fluency, flexibility, and originality represent related but distinguishable components of DT (Guilford 1967; Reiter‐Palmon et al. 2019). If raters' overall evaluations were partly influenced by fluency and flexibility, then participants' ability to predict those dimensions could contribute to above‐chance prediction of overall creativity, even in the absence of accurate originality prediction. Future studies should examine the weighting relationship among overall creativity, fluency, flexibility, and originality more directly, for example by modeling how each dimension contributes to overall creativity ratings.

### 4.4 Alternative Explanations for the Failure to Predict Originality

Although the representational‐transformation account provides one plausible explanation for the failure to predict originality, alternative explanations should also be considered. First, a measurement‐related explanation is possible. In the present study, originality scores were based on the relative frequency of responses within the sample's response pool. Frequency‐based scoring is a common approach in AUT research, as originality is often operationalized as the rarity or infrequency of a response (Guilford 1967; Reiter‐Palmon et al. 2019). However, the present response pool was relatively small. As a result, originality scores may have contained more measurement noise than fluency or flexibility. This issue is especially relevant because recent work has emphasized that originality assessment in the AUT can vary depending on whether responses are scored using subjective ratings, frequency‐based indices, or automated semantic‐distance measures (Beaty and Johnson 2021; Reiter‐Palmon et al. 2019; Vartanian et al. 2020). If originality was measured less reliably or less precisely, prediction accuracy for originality may have been underestimated. Future studies could use larger normative databases, predefined frequency thresholds, consensual assessment procedures, or semantic‐distance methods to obtain more stable originality estimates.

Second, a motivational or strategic explanation should be considered. Participants may have been reluctant to predict high originality because originality is often perceived as difficult, uncertain, or risky. Some individuals may underestimate their own creative originality because of low creative self‐efficacy or conservative self‐evaluation strategies. Creative self‐efficacy refers to individuals' beliefs about their ability to produce creative outcomes and has been shown to relate to creative motivation, creative self‐concept, and creative performance (Beghetto 2006; Karwowski 2016). Such underconfidence could weaken, or even reverse, the association between predicted and actual originality independently of participants' actual metacognitive sensitivity. Future studies could assess creative self‐efficacy, perceived task difficulty, confidence in originality predictions, or willingness to take creative risks to determine whether motivational factors contribute to inaccurate originality prediction.

Third, the specific parameters of the AUT may have shaped the observed pattern. Participants generated verbal responses online within a 2‐min window for each object. A short response window may have favored accessible and conventional responses, potentially constraining the emergence of highly original ideas that often appear later in the ideation sequence. This possibility is consistent with the serial order effect in DT, which shows that idea fluency tends to decline over time whereas originality tends to increase as individuals move beyond obvious responses (Beaty and Silvia 2012; Christensen et al. 1957; Lucas and Nordgren 2020). More recent empirical and review work also supports the view that originality unfolds dynamically across the course of idea generation rather than being evenly distributed throughout the task (Bai et al. 2021; Chen and Ding 2022). In addition, the verbal online format may have introduced individual differences unrelated to creative ability, such as articulation speed, hesitation, background noise, or recording quality. These procedural features may have affected both AUT performance and metacognitive prediction accuracy. Future studies could compare verbal and typed AUT formats, vary response duration, or examine the serial position of ideas to determine whether prediction accuracy changes across early and late stages of idea generation.

### 4.5 Implications for Creative Metacognition

Despite these limitations, the present findings contribute to research on creative metacognition by showing that prospective monitoring accuracy in DT is not uniform across dimensions. Individuals were able to predict dimensions related to idea quantity and category diversity, but not originality. This pattern suggests that creative metacognition should not be treated as a single global ability. Instead, metacognitive prediction may depend on the specific cognitive processes underlying each component of creative performance.

The findings also help refine the boundary between general metacognition and creative metacognition. In memory tasks, prediction accuracy often depends on cue diagnosticity: when subjective cues are valid indicators of later performance, predictions tend to be accurate; when cues are misleading, predictions become inaccurate (Koriat 1997). The present study extends this logic to DT. Fluency and flexibility may be supported by relatively diagnostic cues, whereas originality may rely on cues that are less available or less diagnostic before task performance. Thus, models of creative metacognition should distinguish between monitoring processes that are useful for predicting accessible, retrieval‐based aspects of creativity and those required for monitoring originality, which may involve later‐emerging and less predictable transformations.

Practically, the inability to predict originality may have important consequences for creative self‐regulation. If individuals rely too heavily on early feelings of fluency, they may undervalue tasks or ideas that initially feel difficult but later yield original responses. This could lead to premature disengagement from creative work. Creativity training may therefore benefit from helping individuals distinguish between ease of idea generation and originality, and from teaching them that original ideas may emerge after common responses have been exhausted.

### 4.6 Limitations and Future Directions

Despite its contributions, the present study has several limitations. First, although the 30‐s prediction window was designed to constrain covert idea generation during the prediction stage, future research should include more direct manipulation checks, such as thought probes, to verify whether participants generated possible uses while making their predictions. In addition, because the prediction stage was implemented as a verbal ranking task rather than a computerized trial‐by‐trial prediction task, reaction times for individual ranking decisions were not recorded. Future studies could use computerized item‐by‐item prediction procedures to examine not only prediction accuracy but also the time course and consistency of metacognitive prediction. The online verbal AUT format may also have introduced variability related to articulation speed, background noise, or recording accuracy; laboratory‐based typed responses may reduce such noise in future studies.

Second, the present study did not formally assess or control for object familiarity, semantic association breadth, or visual complexity, all of which may influence both AUT performance and metacognitive prediction accuracy. Future work should systematically manipulate or match object characteristics to test whether these variables mediate prediction accuracy. In addition, detailed information about participants' academic majors or fields of study was not collected, which limits our ability to examine whether educational background influenced AUT performance or metacognitive prediction accuracy.

Third, originality was scored using a relative frequency‐based judgment procedure rather than fixed percentage thresholds. Although this approach is commonly used in AUT scoring and allowed raters to evaluate the rarity of responses within the present response pool, the absence of predefined frequency cutoffs may have introduced some subjectivity into originality ratings. Future studies should consider using larger normative databases or explicitly defined frequency thresholds to improve the precision and comparability of originality scoring.

Finally, the present design focused on item‐level prediction. Although this approach allowed us to examine whether individuals could predict which AUT objects would elicit better performance, it did not capture how metacognitive monitoring unfolds during idea generation. Future studies could examine trial‐level or idea‐level predictions, post‐idea confidence judgments, and serial‐order changes in originality to provide finer‐grained insights into the dynamics of creative metacognition.

## 5 Conclusion

In conclusion, the present study demonstrates that individuals can prospectively predict some, but not all, aspects of their DT performance. Prediction accuracy was above chance for fluency and flexibility, but not for originality. These findings suggest that metacognitive prediction in DT is dimension‐specific: individuals may rely on accessibility‐based cues to anticipate idea quantity and category diversity, but these cues may not support accurate prediction of originality. By distinguishing prediction accuracy across components of DT, the present study refines theories of creative metacognition and highlights the need to examine not only whether people can monitor creativity, but also which aspects of creativity they can monitor accurately.

## Funding

This work was supported by the National Natural Science Foundation of China (32271097, 31671124, 31871093).

## Conflicts of Interest

The authors declare no conflicts of interest.

## Data Availability

The datasets used and/or analysed during the current study are available from the corresponding author on reasonable request.
